# Can FSH influence longevity?

**DOI:** 10.1111/acel.12663

**Published:** 2017-08-30

**Authors:** Andrzej Bartke

**Affiliations:** ^1^ Department of Internal Medicine SIU School of Medicine 801 N. Rutledge, P.O. Box 19628 Springfield IL 62704‐9628 USA

**Keywords:** Aging, life span, longevity, longevity regulation, mice, molecular biology of aging

## Abstract

It was recently reported that the extragonadal actions of follicle‐stimulating hormone (FSH) include regulation of brown and white adipose tissue function and thermogenesis. Based on these findings and on our evidence for reduced FSH levels and enhanced thermogenesis in long‐lived growth hormone (GH)‐deficient mice and GH‐resistant mice, we suggest that FSH may have a role in the control of aging and longevity. We speculate that alterations in FSH secretion may represent one of the mechanisms of trade‐offs between reproduction and aging.

Recent evidence for extragonadal actions of follicle‐stimulating hormone (FSH), including effects on the function of both brown and white adipose tissue (Liu *et al*., 2017), raises the intriguing possibility that FSH may be involved in the control of aging. If confirmed, this novel action of FSH would enhance our understanding of mechanisms and trade‐offs involved in the control of healthspan and longevity of homeothermic organisms.

Follicle‐stimulating hormone is produced by the anterior pituitary gland and acts as one of the master regulators of reproductive functions in both females and males. Recent work indicates that FSH also acts within nonreproductive tissues, including bone (Sun *et al*., 2005). In a paper recently published in *Nature* (Liu *et al*., 2017), Liu *et al*. provide elegant evidence that FSH also influences adipose tissue functions; specifically, blocking FSH actions with a specific antibody‐stimulated thermogenesis in both brown and white adipose tissues (BAT and WAT), reduced adiposity, and increased bone mass in laboratory mice. These findings imply that the well‐documented postmenopausal increase in FSH secretion is likely among the causes for increased adiposity, reduction in bone mass, and alterations in energy metabolism at this stage of life history. One could also suspect that the gradual age‐related increase in FSH levels in men has similar consequences.

Extrapolating from the findings of Liu *et al*. (2017), we suggest that the physiological changes resembling the benefits of blocking FSH action may also occur in response to a modest reduction in FSH levels in animals genetically predisposed to extreme longevity. This would imply that FSH may have a role in the control of aging. Our hypothesis that FSH may have a role in the control of aging stems from observations in two types of mice in which reduction in FSH levels, activation of BAT, browning of WAT, and increased energy expenditure are associated with major extensions of healthspan and longevity. In *Prop1* ^*d*f^ mutants with a genetic defect in the differentiation of somatotroph, lactotroph, and thyrotroph cell lineages in the anterior pituitary, the expression of the FSH‐β subunit gene, the pituitary FSH content, and the plasma FSH levels are significantly reduced (Tang *et al*., 2017). A similar reduction in plasma FSH levels occurs in both sexes of mice with deletion of the growth hormone receptor (GHR) gene (Chandrashekar *et al*., 2007, 2004). In both *Prop1* ^*df*^ (Ames dwarf) and GHR−/− (Laron dwarf) mice, BAT is enlarged and highly active (Li *et al*., 2003; Darcy *et al*., 2016), subcutaneous WAT exhibits characteristics of ‘beiging’ (unpublished data) and metabolic rate (measured by the rate of oxygen consumption per unit of body mass) is increased during both active (dark) and resting (light) phases of the diurnal cycle (Westbrook *et al*., 2012). Moreover, their respiratory exchange ratio is reduced, implying a shift in mitochondrial function toward greater utilization of fatty acids as metabolic fuel (Westbrook *et al*., 2012). Both Ames dwarf and GHR−/− mice are remarkably long‐lived, with increases in longevity ranging from some 20% to over 60% depending on the diet, gender, and genetic background of the animal (Brown‐Borg *et al*., 1996; Coschigano *et al*., 2003; Bartke *et al*., 2013). The enhancement of longevity in these mice is associated with maintenance of youthful levels of cognitive and neuromuscular function, and other phenotypic characteristics into advanced age (Bartke *et al*., 2013), delayed onset, and reduced incidence of cancer and other age‐related pathological changes (Ikeno *et al*., 2009; Bartke *et al*., 2013), and a reduced rate of aging (Koopman *et al*., 2016). We have previously proposed that the activation of BAT and increased metabolic rate in these long‐lived mice represent responses to increased radiational heat loss in these diminutive animals (Westbrook, 1993; Darcy *et al*., 2016). However, results of GH replacement therapy in *Prop1* ^*df*^ mice indicate that their metabolic characteristics and extended longevity can be at least partially uncoupled from body size (Sun *et al*., 2006). Extrapolating from the recent findings of Liu *et al*. (2017), we now hypothesize that reduced FSH levels in these animals may produce (or contribute to) their unique metabolic profile, and also likely to their extended longevity.

If confirmed, the effects of reduced FSH levels in these animals would represent a novel mechanism of trade‐offs between their fertility and longevity. Both sexes of GHR−/− mice and male Ames dwarfs are fertile, but their sexual maturation is delayed and fecundity is reduced (Bartke *et al*., 2013). Important trade‐offs between reproduction and aging have been studied and discussed for decades (Bartke *et al*., 2013), but our understanding of the underlying mechanisms is very limited, and in mammals, virtually nonexistent. Thus, combining the results of FSH blockage reported by Liu *et al*. (2017), Sun *et al*. (2006) with the available information on endocrine and metabolic characteristics of two types of long‐lived mice produces a novel mechanistic insight into the complex interaction of sexual maturation, reproductive effort, fecundity, aging, and lifespan.

## Funding

No funding information provided.

## Conflict of interest

None declared.

## References

[acel12663-bib-0001] Bartke A , Sun LY , Longo V (2013) Somatotropic signaling: trade‐offs between growth, reproductive development, and longevity. Physiol. Rev. 93, 571–598. 23589828 10.1152/physrev.00006.2012 PMC3768106

[acel12663-bib-0002] Brown‐Borg HM , Borg KE , Meliska CJ , Bartke A (1996) Dwarf mice and the ageing process. Nature 384, 33. 10.1038/384033a0 8900272

[acel12663-bib-0003] Chandrashekar V , Dawson CR , Martin ER , Rocha JS , Bartke A , Kopchick JJ (2007) Agerelated alterations in pituitary and testicular functions in long‐lived growth hormone receptor gene‐disrupted mice. Endocrinology 148, 6019–6025. 17872367 10.1210/en.2007-0837

[acel12663-bib-0004] Chandrashekar V , Zaczek D , Bartke A (2004) The consequences of altered somatotropic system on reproduction. Biol. Reprod. 71, 17–27. 15028633 10.1095/biolreprod.103.027060

[acel12663-bib-0005] Coschigano KT , Holland AN , Riders ME , List EO , Flyvbjerg A , Kopchick JJ (2003) Deletion, but not antagonism, of the mouse growth hormone receptor results in severely decreased body weights, insulin, and insulin‐like growth factor I levels and increased life span. Endocrinology 144, 3799–3810. 12933651 10.1210/en.2003-0374

[acel12663-bib-0006] Darcy J , McFadden S , Fang Y , Huber JA , Zhang C , Sun LY , Bartke A (2016) Brown adipose tissue function is enhanced in long‐lived, male ames dwarf mice. Endocrinology 157, 4744–4753. 27740871 10.1210/en.2016-1593 PMC5133358

[acel12663-bib-0007] Ikeno Y , Hubbard GB , Lee S , Cortez LA , Lew CM , Webb CR , Berryman DE , List EO , Kopchick JJ , Bartke A (2009) Reduced incidence and delayed occurrence of fatal neoplastic diseases in growth hormone receptor/binding protein knockout mice. J. Gerontol. A Biol. Sci. Med. Sci. 64, 522–529. 19228785 10.1093/gerona/glp017 PMC2667132

[acel12663-bib-0008] Koopman JJ , van Heemst D , van Bodegom D , Bonkowski MS , Sun LY , Bartke A (2016) Measuring aging rates of mice subjected to caloric restriction and genetic disruption of growth hormone signaling. Aging 8, 539–546. 26959761 10.18632/aging.100919 PMC4833144

[acel12663-bib-0009] Li Y , Knapp JR , Kopchick JJ (2003) Enlargement of interscapular brown adipose tissue in growth hormone antagonist transgenic and in growth hormone receptor gene‐disrupted dwarf mice. Exp. Biol. Med. 228, 207–215. 10.1177/153537020322800212 12563029

[acel12663-bib-0010] Liu P , Ji Y , Yuen T , Rendina‐Ruedy E , DeMambro VE , Dhawan S , Abu‐Amer W , Izadmehr S , Zhou B , Shin AC , Latif R , Thangeswaran P , Gupta A , Li J , Shnayder V , Robinson ST , Yu YE , Zhang X , Yang F , Lu P , Zhou Y , Zhu LL , Oberlin DJ , Davies TF , Reagan MR , Brown A , Kumar TR , Epstein S , Iqbal J , Avadhani NG , New MI , Molina H , van Klinken JB , Guo EX , Buettner C , Haider S , Bian Z , Sun L , Rosen CJ , Zaidi M (2017) Blocking FSH induces thermogenic adipose tissue and reduces body fat. Nature 546, 107–112. 28538730 10.1038/nature22342 PMC5651981

[acel12663-bib-0011] Partridge L , Gems D , Withers DJ (2005) Sex and death: What is the connection? Cell 120, 461–472. 15734679 10.1016/j.cell.2005.01.026

[acel12663-bib-0012] Sun L , Peng Y , Sharrow AC , Iqbal J , Zhang Z , Papachristou DJ , Zaidi S , Zhu LL , Yaroslavskiy BB , Zhou H , Zallone A , Sairam MR , Kumar TR , Bo W , Braun J , Cardoso‐Landa L , Schaffler MB , Moonga BS , Blair HC , Zaidi M (2006) FSH directly regulates bone mass. Cell 125, 247–260. 16630814 10.1016/j.cell.2006.01.051

[acel12663-bib-0013] Sun LY , Fang Y , Patki A , Koopman JJ , Allison DB , Hill CM , Masternak MM , Darcy J , Wang J , McFadden S , Bartke A (2017) Longevity is impacted by growth hormone action during early postnatal period. Elife 6, e24059. 28675141 10.7554/eLife.24059 PMC5515575

[acel12663-bib-0014] Tang K , Bartke A , Gardiner CS , Wagner TE , Yun JS (1993) Gonadotropin secretion, synthesis, and gene expression in human growth hormone transgenic mice and in Ames dwarf mice. Endocrinology 132, 2518–2524. 8504754 10.1210/endo.132.6.8504754

[acel12663-bib-0015] Westbrook R (2012). The effects of altered growth hormone signaling on murine metabolism. Ph.D. Dissertation, Southern Illinois University, Carbondale.

[acel12663-bib-0016] Westbrook R , Bonkowski MS , Strader AD , Bartke A (2009) Alterations in oxygen consumption, respiratory quotient, and heat production in long‐lived GHRKO and Ames dwarf mice, and shortlived bGH transgenic mice. J. Gerontol. A Biol. Sci. Med. Sci. 64, 443–451. 19286975 10.1093/gerona/gln075 PMC2657169

